# Domestication of azuki bean and soybean in Japan: From the insight of archeological and molecular evidence

**DOI:** 10.1270/jsbbs.22074

**Published:** 2023-04-27

**Authors:** Yu Takahashi, Hiroo Nasu, Seiji Nakayama, Norihiko Tomooka

**Affiliations:** 1 Research Center of Genetic Resources, National Agriculture and Food Research Organization, Tsukuba, Ibaraki 305-8602, Japan; 2 Faculty of Biosphere-Geosphere Science, Okayama University of Science, Okayama 700-0005, Japan; 3 Research Institute of Cultural Properties, Teikyo University, Fuefuki, Yamanashi 406-0032, Japan

**Keywords:** azuki bean, domestication, soybean

## Abstract

Domestication of azuki bean and soybean has enabled them to acquire non-dormant seeds, non-shattering pods, and larger seed size. Seed remains of the Jomon period recently discovered at archeological sites in the Central Highlands of Japan (6,000–4,000 BP) suggest that the use of azuki bean and soybean and their increase in seed size began earlier in Japan than in China and Korea; molecular phylogenetic studies indicate that azuki bean and soybean originated in Japan. Recent identification of domestication genes indicate that the domestication traits of azuki bean and soybean were established by different mechanisms. Analyses of domestication related genes using DNA extracted from the seed remains would reveal further details about their domestication processes.

## Domestication traits of legume crops

Crop plants have been established through the domestication of wild plants. Wild plants have been bred into their current crop phenotypes over thousands of years through repeated cultivation and selection by humans. Abbo *et al.* (2014) defined domestication as follows: “In biological terms, domestication refers to the major genetically based phenotypes characterizing plants selected by humans. In cultural terms, domestication is an episode based on a decision and follow-up action by which humans have chosen certain species and selected particular stocks for growth. Thus, domestication involves obtaining desirable plants with distinct phenotypes by taking educated and conscious decisions”. The “phenotypic traits characterizing plants selected by humans” in this definition are generally referred to as domestication traits. In legumes, the primary domestication traits are seed dormancy, pod shattering, and seed size (Abbo *et al.* 2014, Fuller and Allaby 2009, Smýkal *et al.* 2014).

### Seed dormancy

**Fig. 1.**
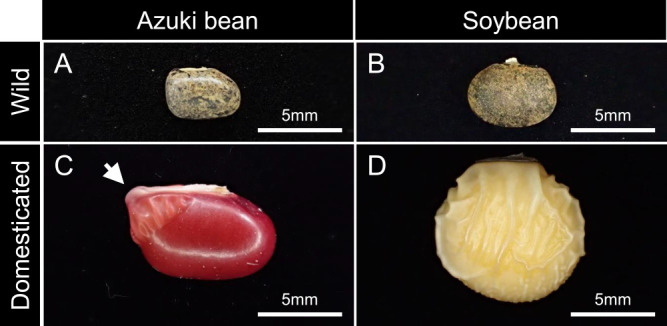
Differences in seed water absorption mechanisms acquired by domesticated azuki bean and soybean. Most seeds of (A) wild azuki bean and (B) wild soybean do not absorb water even when submerged in water. (C) In domesticated azuki bean with red seed coat, water absorption starts from a specialized tissue known as lens (indicated by the arrow). (D) Domesticated soybean with translucent seed coats can absorb water throughout the seed coat.

While wild legume seeds exhibit seed dormancy, domesticated legumes produce non-dormant seeds. Dormant seeds refer to the phenotype in which the germination of mature seeds is suppressed. For example, in temperate zones with cold winter, summer annuals produce seeds in autumn, but the seeds enter dormancy and do not germinate until spring. However, winter annuals produce seeds in the spring, which remain dormant during summer before germinating in the fall. Dormancy break occurs owing to the loss of seed coat water impermeability or due to environmental signals such as low or high temperatures (Baskin and Baskin 2004). The seeds of several wild legumes exhibit seed coat water impermeability when they reach maturity; this physical dormancy is the result of controlling the absorption of water into the seed. As time passes, a specialized water gap tissue opens up to allow the absorption of water into the seed (Baskin and Baskin 2004) (Fig. 1). Seed dormancy is an important trait that allows the germination of seeds at the right time in their natural environments, but it is an inconvenient trait in crops cultivated and harvested by farmers. However, it is believed that the early farmers did not knowingly select non-dormancy (Purugganan 2019). For example, if a non-dormant mutant occurs in a population through cultivation, it would germinate and be harvested preferentially each year. Therefore, if a population that includes a few non-dormant mutant individuals is cultivated a dozen or so times, it would naturally be dominated by the descendants of non-dormant mutants regardless of whether the cultivator is aware (Ladizinsky 1987). Ladizinsky (1987) proposed a theory that legume crop domestication differed significantly from cereal crop domestication. He argued that lentils had already acquired non-dormancy before they were cultivated, when their seeds were collected from wild populations. Moreover, Zohary (1989) argued that there was not much difference between the domestication of legumes and cereal crops and that legume crops acquired non-dormancy only after they were cultivated.

### Pod shattering

**Fig. 2.**
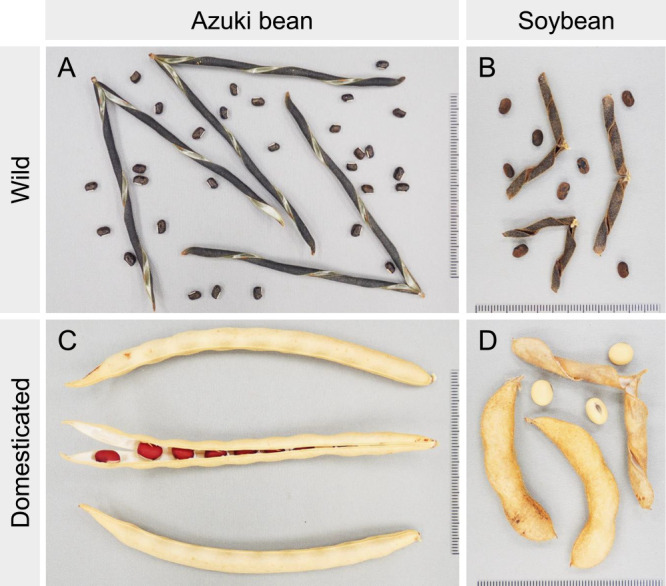
Pods and seeds of wild and domesticated azuki bean and soybean. Upon maturation, (A) wild azuki bean and (B) wild soybean pod valves twist into a helical shape, causing the seeds to scatter. (C) Domesticated azuki bean with the acquired non-shattering pods with pod valves that do not twist. (D) The non-shattering trait of domesticated soybean is inefficient; the helical tension of pod valves remains strong when they dry, making them more likely to shatter than the pods of domesticated azuki bean.

The seeds of legumes are enclosed in pods. When wild legumes reach maturity, the chiral pod valves twist into a helical shape to scatter the seeds (Fig. 2, Armon *et al.* 2011). The pod valves in legumes form from the thick sclerenchyma with a bilayer structure on the endocarp. Cellulose microfibrils in the inner and outer layers of this bilayer structure are located at an angle of approximately ±45° to the longitudinal axis of the pods. When the pods dry, they shrink perpendicularly, twisting the pod valves into a helical shape (Erb *et al.* 2013). The force generated during twisting help scatter the seeds as far as 7 m (Yoshimura *et al.* 2011). If the pods of cultivated crops were to be shattered before harvest, it would be difficult for farmers to harvest their seeds. It is likely that non-shattering pods were unconsciously selected during domestication.

### Seed size

The seeds of legume crops are generally larger than those of their wild ancestors (Figs. 1, 2). Not all seeds produced by wild plants survive under natural environmental conditions; wild plants avoid the risk of extinction by producing a large number of seeds, even if this comes at the expense of seed weight (Šerá and Šerý 2004). Moreover, farmers bury the seeds of their crops while sowing in the soil. This practice reduces the number of seeds eaten by birds or animals and improves rooting. It is believed that burying crop seeds in the soil led to the unconscious selection of larger seeds (Kluyver *et al.* 2013, Purugganan and Fuller 2009), because larger seeds are better at lifting the soil and germinating (Benvenuti *et al.* 2001, Bond *et al.* 1999, Pearson *et al.* 2002). Additionally, there are many examples of conscious selection of larger seeds owing to factors such as appearance and mouthfeel.

## Archeology

Wild ancestors of domesticated azuki bean are found in Japan, southern China, Korea, northern Laos, northern Myanmar, Bhutan, Nepal, and the Indian Himalayas; wild ancestors of domesticated soybean are found in Japan, China, Korea, Taiwan, and the Russian Far East (Lu 2005, Ohashi 2001). Wild azuki bean and wild soybean are characteristic of dormant seeds, shattering pods, and small seeds (Figs. 1, 2).

**Fig. 3.**
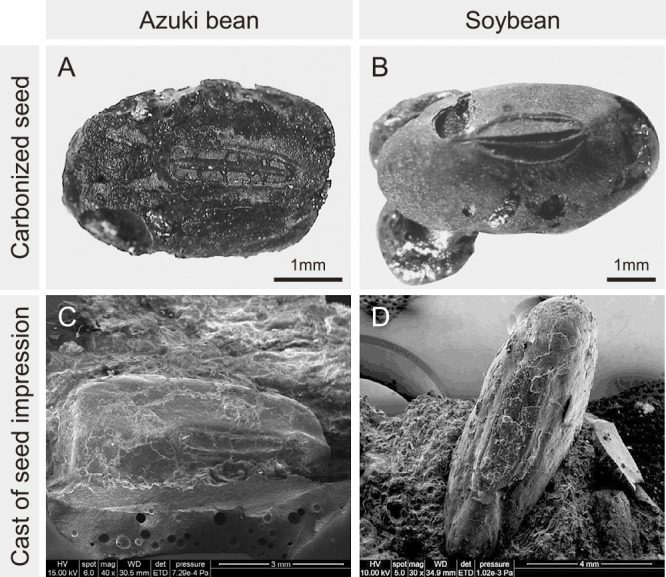
Carbonized seeds and replicas of seed impressions on pottery excavated from archeological sites. (A) Carbonized azuki bean seeds (Kamihara site, unpublished). (B) Carbonized soybean seeds (Shimoyakebe site, 4933 BP, site 18 in Fig. 5 and Table 2). (C) Replicas of azuki bean impressions on pottery created by injecting silicone into the impression (Sakenomiba site, 5180 cal. BP, site 45 in Fig. 5 and Table 1). (D) Replicas of soybean impressions on pottery created by injecting silicone into the impression (Sakenomiba site, 5040 cal. BP, site 45 in Fig. 5 and Table 2).

**Fig. 4.**
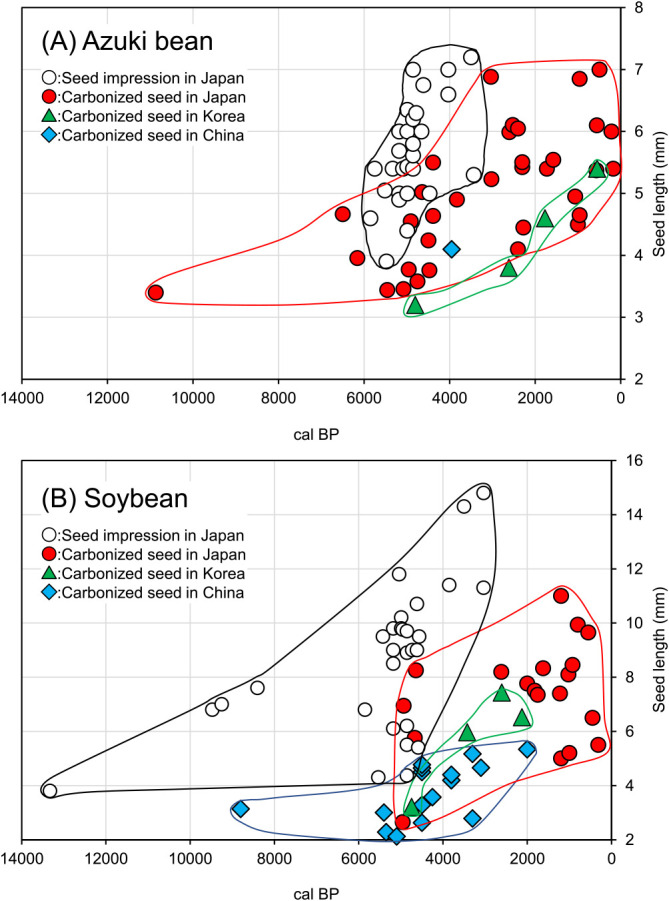
Historical changes in the length (size) of carbonized seeds and replicas of seed impressions on pottery excavated from sites in Japan, Korea, and China. (A) Azuki bean and (B) soybean. The data from China and Korea are only from carbonized seeds. For the azuki bean, the figure was created using 62 data points from Japan (34 carbonized seeds, 28 replica seeds), 1 from China, and 4 from Korea. For soybean, the figure was created using 48 data points from Japan (19 carbonized seeds, 29 replica seeds), 17 from China, and 4 from Korea. The oldest excavated azuki bean and soybean were both from sites in Japan. The diversification of seed sizes that accompanied seed size increase first occurred in Japan. In particular, a large number of replicas of larger azuki bean and soybean seeds were obtained from impressions of pottery from sites in the Central Highlands of Japan, where many sites of Jomon period appeared in the period from 6,000 to 4,000 BC.

While the previous leading theory states that both azuki bean and soybean were domesticated in China, recent findings suggest that wild soybean and wild azuki bean have been domesticated and cultivated in Japan for over 10,000 years. Remains of legume seeds excavated from archeological sites occur as carbonized seeds or seed impressions on pottery. Carbonized seeds refer to the carbonized remains of seeds that have been heated, for example, in a house hearth (Fig. 3A, 3B). Seed impressions on pottery were created when the clay used to make the pot contained seeds; the seeds burned away when the pots were fired, leaving impressions on the outer or inner surfaces of the pots. Casts of seed impressions on pottery are made using a silicone compound to study their morphology and surface structure (Fig. 3C, 3D, Ushino and Tagawa 1991). While seeds shrink when they are burned, the impressions sometimes indicate enlarged seeds owing to the absorption of water during pot making or dried before firing (Nakayama 2009, Nasu *et al.* 2015b, Obata 2011). Therefore, the seed impressions on pottery do not precisely indicate seed size. For example, in a firing experiment investigating wild soybean seed impressions on pottery, there was no difference between the lengths of the original seeds and those of their impressions, whereas in the case of wild azuki bean seeds, the length of the seed impressions was 112% of the original seeds (Nasu *et al.* 2015b). In contrast, with domesticated soybean, the length of the seed impressions was 133% of the original seeds, and with domesticated azuki bean, the length of the seed impressions was 107% of the original seeds. These results indicated that the changes in the size of seed impressions depend on the species (azuki bean or soybean) as well as on whether the seeds exhibit dormancy (wild or domesticated). Additionally, because of the possibility that immature seeds and seeds that have already absorbed water have been embedded in the pots, the figures in this section illustrating the lengths of carbonized seeds and seed impressions on pottery were created using uncorrected data (Fig. 4). Presently, the only domestication trait that can be inferred from carbonized seeds and seed impressions on pottery is the seed size. However, once the genes responsible for non-dormant seeds and non-shattering pods are identified, it would be possible to infer such traits from the carbonized seeds.

**Fig. 5.**
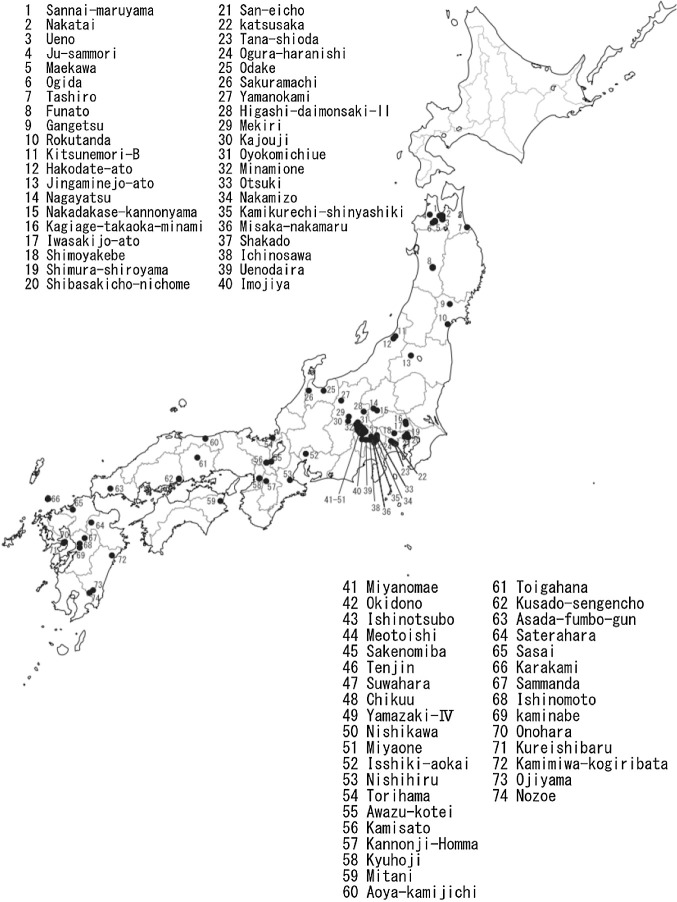
Names of geographical distribution sites in Japan where azuki bean and soybean findings (carbonized seeds or seed impressions on pottery) were excavated.

**Fig. 6.**
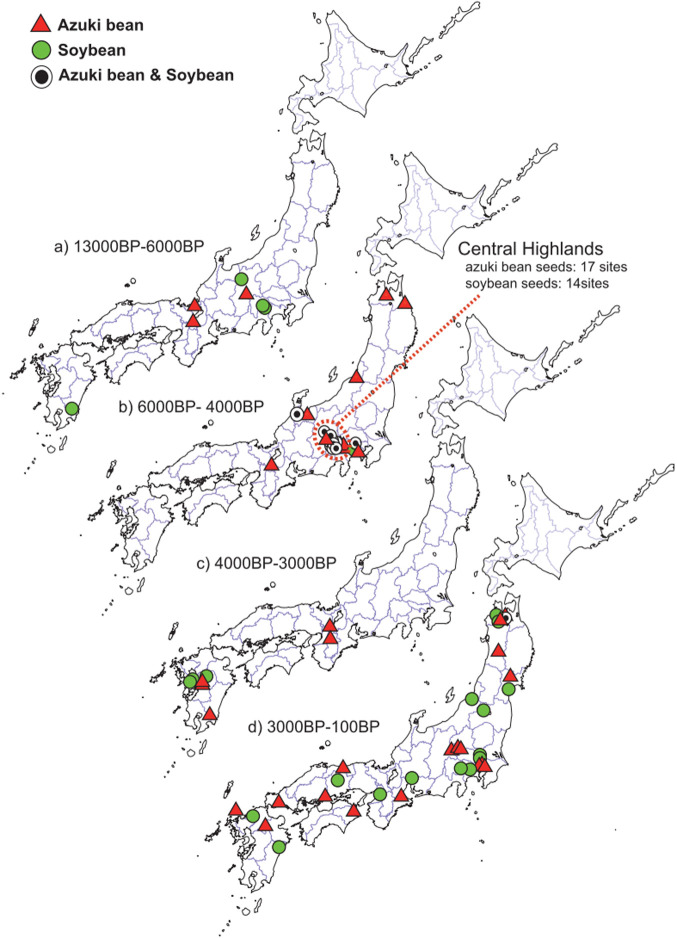
Historical changes in the geographical distribution sites in Japan where azuki bean and soybean findings (carbonized seeds or seed impressions on pottery) were excavated. (A) 13,000–6,000 years ago: no excavations from the Kanto region to the Tohoku region (northern part of Japan). (B) 6,000–4,000 years ago: during a period when the climate was relatively mild, the number of sites in the Central Highlands (Yamanashi and Nagano prefectures) increased sharply, with azuki bean being found even in the northern Tohoku region. (C) 4,000–3,000 years ago: changes in vegetation owing to a rapid cooling event occurring approximately 4,200 years ago have temporarily reduced the quantity of legumes in the Central Highlands and Tohoku region. (D) 3,000–100 years ago: around 3,000 years ago, rice and grain (millet) farming were introduced into Japan from China. Azuki bean and soybean seeds were found in sites throughout Japan excluding Hokkaido Island. The spread of agriculture in the continent could be related to the distribution of azuki bean and soybean throughout Japan.

**Fig. 7.**
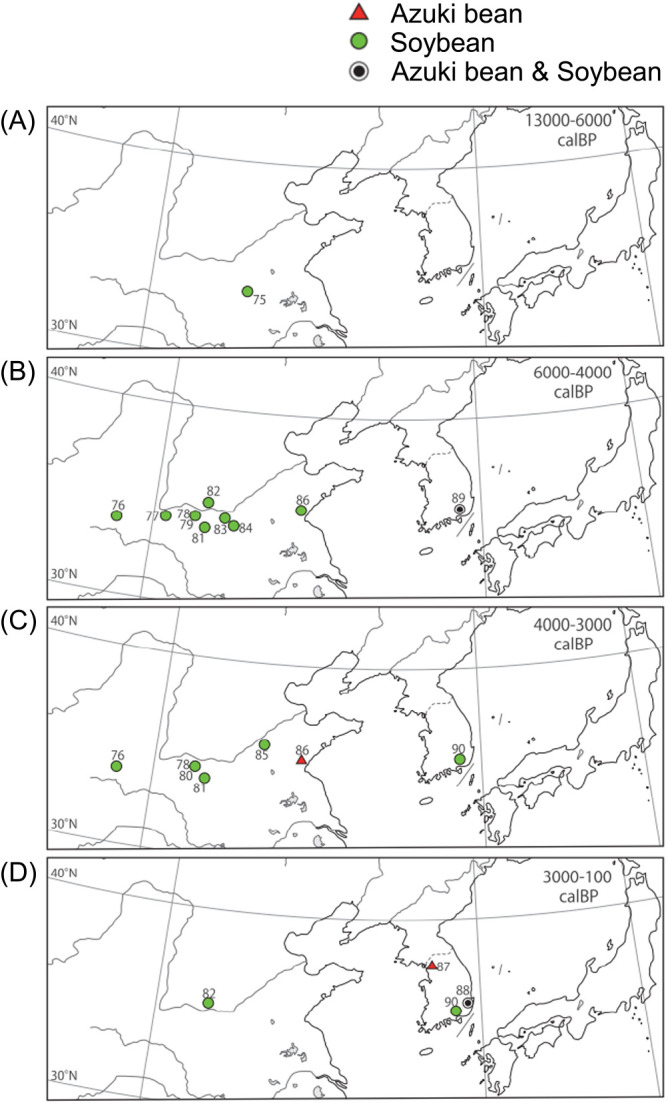
Historical changes in the geographical distribution sites where azuki bean and soybean findings (carbonized seeds) were excavated in China and Korea. The figure was created using all available data on azuki bean and soybean excavation sites. (A) 13,000–6,000 years ago: soybean seeds were found at a single site in China. (B) 6,000–4,000 years ago: in China, the number of sites with soybean increased in the middle reaches of the Yellow River; in Korea, azuki bean and soybean seeds were found in sites toward the south (site 89). (C) 4,000–3,000 years ago: azuki bean seeds were found in China (site 86). (D) 3,000–100 years ago: data from three archeological sites in Korea.

Data on the names and ages of the archeological sites where azuki bean and soybean seed sizes (lengths) were recorded are shown in Tables 1 and 2, respectively. Based on this data, the relationships between the ages of the archeological sites (median of estimated range) and mean sizes (lengths) of the excavated seeds are shown in Fig. 4, the names and locations of sites in Japan where azuki bean and soybean seeds were excavated are shown in Fig. 5, the sites in Japan where azuki bean and soybean seeds were excavated are shown by age in maps in Fig. 6, and the archeological sites in China and Korea where azuki bean and soybean seeds were excavated are shown by age in maps in Fig. 7.

### Archeology of azuki bean

Detailed archeological surveys carried out recently have discovered many carbonized seeds and pottery impressions of azuki bean in Japan (Table 1). The oldest azuki bean sample in Japan, a 3–4 mm carbonized seed dating back to approximately 10,000 years, was excavated from the Awazu lakebed in Shiga Prefecture (Table 1, site 55 in Fig. 5). As the carbonized seeds appear to be approximately of the length as that of the modern wild azuki bean, it is believed that prehistoric people who lived there used wild azuki bean (Table 1, Fig. 4, Minaki and Nakagawa 2000). The high number of archeological records in the period from approximately 6,000 to 4,000 years ago indicates a more intensive use of azuki bean during that period (Figs. 4A, 6B). Particularly, azuki bean seeds have been excavated from many sites in the Central Highlands (Koshin region: Yamanashi and Nagano prefectures) in Japan (Fig. 6B). Increased seed size was observed in both carbonized seeds and seed impressions on pottery during this period (Fig. 4A). Several seed impression data depicting mean seed lengths indicate a seed size (up to 7.00 mm, Table 1) larger than those of modern wild species after absorbing water (5.37 mm, Nasu *et al.* 2015b).

Interestingly, archeological records show that the use of azuki bean declined dramatically in the Central Highlands around 4,000 to 3,000 years ago (Fig. 6C). Instead, large carbonized azuki bean seeds and seed impressions on pottery were found in western Japan and Kyushu Island around this time (Table 1, Figs. 4A, 6C). Although the reason for this remains unclear, it has been suggested that a period of rapid cooling around 4,200–3,800 years ago (Mayewski *et al.* 2004) led to the decrease in chestnut and legume harvests in the Central Highlands, causing humans to migrate and spread the culture of legume cultivation to Kyushu Island located in southern Japan (Nasu 2018, Obata 2016). It is an interesting question whether the large beans that appeared in Kyushu Island around this time had come from the Central Highlands or were brought from Korea or China. Archeological records show the widespread use of azuki bean in Japan around 3,000 years ago (Fig. 6D). Moreover, rice and millet farming were introduced into Japan from China during this period (Nasu and Momohara 2016). Another interesting question is whether the widespread use of azuki bean in Japan (excluding Hokkaido Island) was related to the spread of rice and millet cultivation introduced from China.

Apart from the report by Lee (2013), there are fewer records of azuki bean in Korea than those found in Japan. Presently, the oldest record of azuki bean in Korea include carbonized seeds from the Middle Chulmun period (about 4,800 years ago) excavated from the Pyeonggeodong site (Table 1, site 89 in Fig. 7B). The mean length of these seeds is 3.2 mm (max 4.1 mm), which indicates that the size of azuki bean seeds in Korea around that time was still close to the size of modern wild azuki bean. Increase in seed size appears to have occurred during the Middle Mumun period, approximately 2,600 years ago. The mean seed size during this period was still small (3.8 mm), but some seeds were found to be over 7 mm (Table 1, site 88 in Fig. 7D). After this period, a gradual increase in seed size to a mean size of 4.6 mm has been observed during the Three Kingdoms period (about 1,700 years ago) and 5.4 mm during the Joseon period (approximately 500 years ago) (Table 1, Fig. 4A, site 87 in Fig. 7D). The increase in the size of azuki bean seeds occurred later in Korea than in Japan. Rice and millet farming were introduced into Japan from Korea via northern Kyushu Island around 3,000 years ago. The genetic relationship between the large azuki bean seeds excavated from Korea and those excavated from Japan would be an interesting topic to explore in future studies.

Although there are records of excavated azuki bean in China, there is only one report on their seed size (Table 1, Fig. 4A, site 86 in Fig. 7C). Starch grains from the seeds of species belonging to the tribe Phaseoleae found at the Shizitan site in Shaanxi Province (approximately 13,800–8,500 cal. BP) are believed to be those of the genus *Vigna* (Liu *et al.* 2013). Carbonized azuki bean seeds dating back to approximately 4,000 years were found at the Lianchengzhen site in Shandong Province (4,060–3,840 cal. BP) (Table 1, site 86 in Fig. 7C) (Crawford *et al.* 2005). The maximum seed length was 4.2 mm, which is about the same as that of modern wild azuki bean seeds (Fig. 4A). These findings suggested that azuki bean was not as commonly used in China as in Japan or Korea.

### Archeology of soybean

The oldest soybean reported in Japan is a seed impression on pottery from the Ojiyama site in Miyazaki Prefecture dating back to 13,000 years (Table 2, site 73 in Fig. 5). The seed is 3.8 mm long, equivalent to the size of the modern wild ancestor of soybean (Table 2, Fig. 4B, Obata and Manabe 2012). Reliable evidence collected from approximately 9,000-year-old sites in the Central Highlands (Nagano and Yamanashi prefectures) indicated 6.8–7.6-mm long seed impressions and 4-mm long carbonized seeds discovered in 2009 and 2013, respectively (Table 2, Fig. 4B, sites 27 & 35 in Fig. 5). Similar to the azuki bean, archeological evidence showed the presence of soybean at many archeological sites beyond 6,000 years, mainly in the Central Highlands; some seed impressions on pottery were found to be longer than those of the modern wild ancestors after absorbing water (10 mm) (Fig. 4B). Around this time, seeds with approximately 2–10 times larger volume than that of their wild ancestors became prominent; different seed shapes were also observed, ranging from flat to thicker oval types, showing increased morphological diversification (Nakayama 2015). However, similar to that of the azuki bean, archeological evidence of soybean in the Central Highlands decreased dramatically around 4,000–3,000 years ago, and larger soybean seeds started to appear in Kyushu Island (Fig. 6C). Around 3,000 years ago when rice and millet cultivation was introduced into Japan from China, soybean seeds were found throughout Japan (Fig. 6D).

In China, soybean seeds from the Neolithic period were found in excavations (Lee *et al.* 2011); carbonized seeds from approximately 9,000 years ago were found at the Jiahu site in Henan Province. These seeds were small, measuring an average 3.1 mm length (Table 2, Fig. 4B, site 75 in Fig. 7A). Soybean seeds dating back to 6,000–4,000 years ago were mainly found in the Yellow River Basin (Fig. 7B). During the Longshan cultural period around 4,500 years ago, mean soybean seed length increased to 4.5 mm; during the Shang Dynasty around 3,000 years ago, the mean seed length from site 81 was >5 mm (Table 2). Increasing soybean seed size was observed in China but not as early as that observed in Japan (Fig. 4B).

Carbonized seeds from the Late Chulmun period, i.e., approximately 4,800 years ago, were found at the Pyeonggeodong site in Korea (Table 2, site 89 in Fig. 7B, Lee *et al.* 2011). The mean seed length was 3.2 mm, which is similar to that of the modern wild ancestor (Table 2, Fig. 4B). The mean seed size doubled to approximately 6 mm in the Early Mumun period around 3,500 years ago (Table 2, Fig. 4B, site 90 in Fig. 7C). By the Middle Mumun period around 2,600 years ago, the average seed size exceeded 7 mm (Table 2, site 90 in Fig. 7D), showing a faster rate of seed size increase than that in China (Fig. 4B).

As shown in the examples described above, East Asian archeological records indicate that azuki bean and soybean seed size increased in Japan earlier than in Korea and China. This refutes the conventional theory that azuki bean and soybean have a single origin in China; it could be said that they originated in Japan or have multiple origins, with an earlier origin in Japan. Moreover, the widespread use of azuki bean and soybean in Japan approximately 3,000 years ago indicates the possibility that additional azuki bean and soybean cultivars were introduced from China along with rice and millet. Alternatively, it could be said that only rice and millet were introduced from China, whereas azuki bean and soybean were introduced into Korea and China from Japan. The answers for the above issues could be obtained by integrating archeology and DNA analysis.

## Molecular phylogeny

### Molecular phylogeny of azuki bean

A chloroplast DNA study placed Chinese, Korean, and Japanese domesticated azuki bean in the same group as Korean and Japanese wild azuki bean, whereas wild azuki bean from Nepal, Bhutan, Myanmar, and China were placed in different groups (Ye and Yamaguchi 2008). This suggested that the azuki bean was domesticated in Japan or Korea. Additionally, it has been shown that wild Japanese azuki bean possesses most of the simple sequence repeat alleles of domesticated azuki bean, including those from Bhutan and China (Xu *et al.* 2008). However, their analysis did not include wild Korean azuki bean and included only five samples of wild Chinese azuki bean. Tomooka *et al.* (2014) suggested that Japan is the center of domestication for azuki bean based on the results of DNA analysis (Xu *et al.* 2008, Yamaguchi 1992) as well as on the facts of oldest archeological remains found in Japan. Molecular phylogenetic studies based on resequencing of large-sized samples are yet to be conducted on the azuki bean.

### Molecular phylogeny of soybean

Recent advances in sequencing technology have led to the progress in research on the origins of soybean domestication. Early research on domestication using morphological and seed protein variations suggested that soybean originated in China (Broich and Palmer 1981, Hymowitz 1990, Hymowitz and Kaizuma 1981). Later, a theory of multiple origins in China and Japan emerged based on mutations in chloroplast DNA (Xu *et al.* 2002). The availability of several DNA markers once again supported the single origin theory from China (Guo *et al.* 2010, Li *et al.* 2010). After the release of the complete soybean genome (Schmutz *et al.* 2010), molecular phylogenetic analyses based on resequencing supported the single origin theory (Chung *et al.* 2014, Lam *et al.* 2010, Zhou *et al.* 2015).

Wang *et al.* (2016), who proposed the northern and central Chinese origin theory using high-density single nucleotide polymorphism (SNP) arrays, only analyzed wild and domesticated Chinese soybean accessions. Therefore, their study only investigated regions within China as candidates for the origin for those accessions. In contrast, Jeong *et al.* (2019) analyzed about 4,400 wild and domesticated soybean accessions from Japan, South Korea, North Korea (only domesticated accessions), Far East Russia (only wild accessions), and China using SNP arrays. This study is the most comprehensive investigation to date, and the results suggest that soybean was most likely domesticated only once in eastern Japan.

## Domestication genes

Evolution and domestication are both driven by spontaneous mutations, although the mutations necessary for the acquisition of domestication traits tend to be simpler than those necessary to acquire other evolutionary traits (Meyer and Purugganan 2013). Spontaneous mutations include large structural changes such as chromosome-level insertions, deletions, duplications, inversions, translocations, and reduplications, and smaller mutations include gene-level base substitutions, insertions, and deletions (Crow 1983). Organisms accumulate a variety of spontaneous mutations over long periods of time, where genes and gene networks with new functions are constructed, creating a diversity of living beings. In contrast, domestication was achieved within a span of 12,000 years since humans began farming, which is a relatively short period (Meyer and Purugganan 2013). Recently, genes controlling certain domestication traits in major crops have been discovered. Such domestication genes are often transcription factors that regulate genes, and nonsense mutations that impair certain gene functions are often the primary factors involved in the development of domestication traits (Meyer and Purugganan 2013).

### Domestication genes of azuki bean

The mechanisms through which azuki bean acquired certain domestication traits differ from those of domesticated soybean. Although domesticated soybean with yellow (translucent) seed coat absorbs water throughout its structure, domesticated azuki bean with red seed coat absorbs water only through a specialized tissue known as the lens (Fig. 1) (Kikuchi *et al.* 2006). The differences in mechanisms of absorption of soybean and azuki bean seeds indicated that the water absorption rate of domesticated azuki bean is much slower than that of the domesticated soybean (Uenaka *et al.* 2000). Among the five loci detected for seed dormancy, one of them was tightly linked to seed coat color (red or tan) in azuki bean (Isemura *et al.* 2007). However, genes responsible for seed dormancy of azuki bean are yet to be identified. Although the gene *VaSDC1* encoding a putative R2R3-type MYB transcription factor was identified as the candidate gene for seed coat color (red or black), it has not been associated with seed dormancy (Chu *et al.* 2021).

**Fig. 8.**
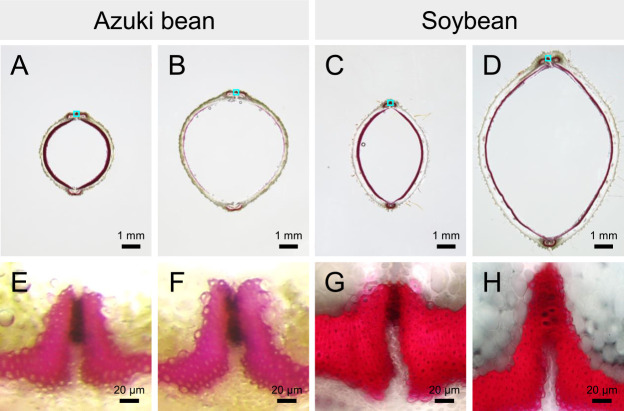
Changes in lignin accumulation patterns in the inner walls and abscission zones of pods, which caused the loss of the pod shattering trait through the domestication of azuki bean and soybean. Both (A) wild azuki bean and (C) wild soybean have a thick lignin layer stained red on the inner wall of the pod. (B) In domesticated azuki bean, the lignin layer of the pod is much thinner than that in wild azuki bean, and the dried pods do not have helical tension. (D) The inner lignin layer of domesticated soybean is similar to that of wild soybean; dried pods still have helical tension. (E–H) An enlarged view of the cyan box in Fig. 8A–8D, respectively. The abscission layer (dark area: the part that splits open the pods to scatter the seeds) is as clear in the pods of (E) wild azuki bean, (F) domesticated azuki bean, and (G) wild soybean. (H) In domesticated soybean, pods have acquired the non-shattering trait from stronger adhesion by excessive lignification of the fiber cap cells connecting the pod valves, but helical tension of the pod valves remains strong, overcoming the adhesion sometimes.

The non-shattering pod phenotype of domesticated azuki bean is superior to that of domesticated soybean. Although wild soybean and azuki bean display the same pod shattering mechanism, i.e., through the helical tension generated on the dried pod (Fig. 2A, 2B), domesticated soybean and azuki bean acquired non-shattering pod traits through different mechanisms. In the case of azuki bean, a phenotypic variation that greatly reduces the helical tension of the pod valves by thinning the lignin layer was selected (Fig. 8A, 8B), and the adhesion of the seam that connects the pod valves did not change between wild and domesticated azuki beans (Fig. 8E, 8F) (Takahashi *et al.* 2020). In contrast, wild and domesticated soybean did not vary in terms of thickness of lignin layer (Fig. 8C, 8D), and the adhesion of the seam that connects the pod valves did change (Fig. 8G, 8H). The non-shattering pod phenotype of domesticated azuki bean is controlled by a single locus (Isemura *et al.* 2007). Fine-mapping results suggest that a loss of function mutation of the *VaMYB26* (Vigan.07g034400) encoding a putative R2R3-type MYB transcription factor is responsible for the thinning of the lignin layer in the pods of domesticated azuki bean (Takahashi *et al.* 2020). The putative *MYB26* orthologs have also been identified as candidate genes for non-shattering pods in common bean (Di Vittori *et al.* 2021).

The mechanisms involved in seed size increase are yet to be clarified in azuki bean. Although six loci involved in seed size differences have been identified between wild and domesticated azuki beans (Isemura *et al.* 2007), the candidate gene is yet to be identified. Future research should focus on the molecular mechanisms involved in seed size increase in azuki bean.

### Domestication genes of soybean

Eight quantitative trait loci (QTL) for seed dormancy were detected between wild and domesticated soybeans (Kuroda *et al.* 2013). Two genes responsible for the control of physical seed dormancy of soybean have been identified to date: (i) *GmHs1-1* (Glyma.02G269500), which encodes a calcineurin-like transmembrane metallophosphoesterase involved in the regulation of calcium content of seed coat (Sun *et al.* 2015); and (ii) *GmGH9B8* (Glyma.02G269400), encoding an endo-1,4-β-glucanase involved in the development of cracks on the seed coat surface (Jang *et al.* 2015). Unlike the two aforementioned genes, the *G* gene (Glyma.01g198500), encoding a CAAX amino-terminal protease protein, affects physiological seed dormancy through interactions with key enzymes that modulate abscisic acid synthesis (Wang *et al.* 2018). The *G* gene also determines differences in seed coat color (yellow or green) among varieties (Wang *et al.* 2018). Additionally, two QTLs responsible for water permeability of the seed coat are tightly linked to the *I* and *T* loci for seed coat color (Kuroda *et al.* 2013). These findings suggest that part of the signaling molecules and secondary metabolites that determine seed coat color may affect physiological as well as physical seed dormancy.

A genome-wide association study on soybean pod shattering identified loci harboring *SHAT1-5*, *Pdh1*, and *NST1A* as loci harboring genes (Zhang and Singh 2020). *SHAT1-5* (Glyma.16G019400) encoding a NAC domain transcription factor was selected during soybean domestication (Dong *et al.* 2014). A gain of function mutation of the *SHAT1-5* makes pods less likely to shatter, because the adhesion of the seam that connects the pod valves is reinforced by excessive lignification, which resists the helical tension of pod valves (Fig. 8G, 8H). A severe selective sweep was detected across the ~116 kb region including *SHAT1-5* on chromosome 16, indicating a strong artificial selection on this gene. The *PDH1* (Glyma16g25580) encoding a dirigent-like protein was found to be associated with controlling the helical tension of the pod valves through modification of the inner sclerenchyma cells (Funatsuki *et al.* 2014). Although *PDH1* was initially identified based on differences among cultivars, its non-shattering allele was found in wild soybean from the Huang-Huai-Hai valleys of China, suggesting that its alleles may have originated owing to natural variation in this region (Zhang and Singh 2020). Recently, a gain of function allele of *NST1A* (Glyma.07g050600), a paralog of *SHAT1-5*, was reported as a possible contributor to the non-shattering pod phenotype (Zhang and Singh 2020); however, the SNP detected by genome-wide association analysis is 49 kb away from the other SNP that gives rise to a premature stop codon in *NST1A*; therefore, additional analysis including transformation are required.

Nineteen QTLs have been detected for seed size differences between wild and domesticated soybean, and two genes have been identified to date (Karikari *et al.* 2019). *PP2C* (Glyma17g33690), which encodes a type-2C protein phosphatase, contributes to seed size increase by enhancing the cell size of the integument and activating a subset of seed trait-related genes (Lu *et al.* 2017). The *ln* (Glyma20g25000), which encodes a zinc finger protein JAGGED, has a significant effect on the number of seeds per pod and seed size (Jeong *et al.* 2012). Owing to the involvement of several loci and their associated effects, albeit small, there is no clarity on how soybean seeds enlarged during domestication.

## Need for DNA archeology

Studies focusing on the domestication genes of azuki bean and soybean are yet to be conducted using DNA from archeological findings. Yano *et al.* (2004) extracted DNA from carbonized legume seed remains dating back to approximately 4,660 years from the Sannai-Maruyama site in Aomori Prefecture, Japan. Based on chloroplast DNA markers, the specimen were identified as azuki bean seeds. If the genes that control domestication traits could be sequenced from carbonized azuki bean and soybean seeds, it should be possible to determine the degree of domestication of the individual plants, for example, whether they had seed dormancy or pod shattering traits. In conclusion, there is a need for close cooperation between archeologists and molecular geneticists to find answers to these questions.

## Author Contribution Statement

YT conceptualized this review. YT, HN, SN and TN wrote the manuscript.

## Figures and Tables

**Table 1.** Azuki bean seeds (carbonized or impression) found in archaeological sites of China, Korea, and Japan

Country	Locality of archaeological site	Site No.*^a^*	Age Median (calBP)	Type of archaeobotanical finds	No. of samples	Mean seed length (mm)	SD of mean seed length	References
China	Lianchengzhen, Shandong	86	3950	Carbonized seed	9	4.10		Lee (2013)
Korea	Pyeonggeodong, South Gyeongsang	89	4805	Carbonized seed	37	3.20	0.30	Lee (2013)
Korea	Daundong, Ulsan	88	2615	Carbonized seed	653	3.80	0.60	Lee (2013)
Korea	Giheung-Gugal, Gyeonggi	87	1770	Carbonized seed	110	4.60	0.80	Lee (2013)
Korea	Giheung-Gugal, Gyeonggi	87	550	Carbonized seed	12	5.40	0.50	Lee (2013)
Japan	Awazu-kotei, Otsu, Shiga	55	10872	Carbonized seed	18	3.40	0.70	Minaki and Nakagawa (2000)
Japan	Torihama, Mikata, Fukui	54	6500	Carbonized seed	9	4.67	0.38	Matsumoto (1979)
Japan	Kajoji, Okaya, Nagano	30	6156	Carbonized seed	9	3.96	0.39	Nasu *et al.* (2015a)
Japan	Yamazaki-IV, Hokuto, Yamanashi	49	5850	Impression	1	4.60		Nakayama and Sano (2014c)
Japan	Odake, Toyama, Toyama	25	5750	Impression	2	5.40	1.41	Obata *et al.* (2014b)
Japan	Uenodaira, Kofu, Yamanashi	39	5515	Impression	2	5.05	1.06	Nakayama (2014b)
Japan	Kitsunemori-B, Shibata, Niigata	11	5475	Impression	1	3.90		Sasaki (2007)
Japan	Sannai-maruyama, Aomori, Aomori	1	5457	Carbonized seed	9	3.44	0.33	Sasaki (2013)
Japan	Tana-shioda, Sagamihara, Kanagawa	23	5330	Impression	1	5.40		Nakayama and Sano (2015)
Japan	Shakado, Fuefuki/Koshu, Yamanashi	37	5180	Impression	1	6.00		Nakayama and Akiyama (2014)
Japan	Imojiya, Minami-Alps, Yamanashi	40	5180	Impression	1	5.00		Nakayama and Hosaka (2014)
Japan	Sakenomiba, Hokuto, Yamanashi	45	5180	Impression	8	5.69	0.67	Nakayama *et al.* (2009)
Japan	Suwahara, Hokuto, Yamanashi	47	5180	Impression	1	4.90		Nakayama and Sano (2014a)
Japan	Ishinotsubo, Nirasaki, Yamanashi	43	5090	Impression	1	5.40		Nakayama and Uruma (2014a)
Japan	Sannai-maruyama, Aomori, Aomori	1	5080	Carbonized seed	31	3.46	0.39	Sasaki (2013)
Japan	Ichinosawa, Fuefuki, Yamanashi	38	4990	Impression	1	5.00		Nakayama (2014d)
Japan	Ishinotsubo, Nirasaki, Yamanashi	43	4990	Impression	4	6.35	0.66	Nakayama and Uruma (2014a)
Japan	Suwahara, Hokuto, Yamanashi	47	4990	Impression	3	5.43	0.58	Nakayama and Sano (2014a)
Japan	Nishikawa, Hokuto, Yamanashi	50	4990	Impression	1	6.00		Nakayama and Sano (2014d)
Japan	Miyaone, Hokuto, Yamanashi	51	4990	Impression	1	4.40		Nakayama and Sano (2014b)
Japan	Mekiri, Okaya, Nagano	29	4955	Carbonized seed	54	3.77	0.63	Aida *et al.* (2012) Nasu *et al.* (2015a)
Japan	Shimoyakebe, Higashi-murayama, Tokyo	18	4908	Carbonized seed		4.55		Kudo and Sasaki (2010)
Japan	Mekiri, Okaya, Nagano	29	4855	Impression	15	5.61	0.87	Aida *et al.* (2012)
Japan	Ishinotsubo, Nirasaki, Yamanashi	43	4855	Impression	1	5.40		Nakayama and Uruma (2014a)
Japan	Meotoishi, Hokuto, Yamanashi	44	4855	Impression	1	6.20		Nakayama and Uruma (2009)
Japan	Suwahara, Hokuto, Yamanashi	47	4855	Impression	1	7.00		Nakayama and Sano (2014a)
Japan	Nishikawa, Hokuto, Yamanashi	50	4855	Impression	1	5.80		Nakayama and Sano (2014d)
Japan	Ishinotsubo, Nirasaki, Yamanashi	43	4780	Impression	1	6.30		Nakayama and Uruma (2014a)
Japan	Awazu-kotei, Otsu, Shiga	55	4750	Carbonized seed	18	3.58		Nakagawa (1997)
Japan	Suwahara, Hokuto, Yamanashi	47	4660	Impression	1	6.00		Nakayama and Sano (2014a)
Japan	Sakuramachi, Koyabe, Toyama	26	4643	Carbonized seed	37	5.02		Obata (2011) Yamada and Tsubakizaka (2009)
Japan	Ishinotsubo, Nirasaki, Yamanashi	43	4615	Impression	2	6.75	1.20	Nakayama and Uruma (2014a)
Japan	Ooyokomichiue, Hara, Nagano	31	4496	Carbonized seed	57	4.24	0.75	Nasu *et al.* (2015a)
Japan	Minamione, Hara, Nagano	32	4473	Carbonized seed	11	3.76	0.42	Nasu *et al.* (2015a)
Japan	Miyanomae, Hokuto, Yamanashi	41	4470	Impression	2	5.00	0.99	Nakayama and Uruma (2014b)
Japan	Tashiro, Hachinohe, Aomori	7	4385	Carbonized seed	19	4.64	0.48	Sasaki (2013)
Japan	Otsuki, Otsuki, Yamanashi	33	4385	Carbonized seed	10	5.50		Matsutani (1997)
Japan	Shimoyakebe, Higashi-murayama, Tokyo	18	4030	Impression	1	6.60		Obata *et al.* (2014a)
Japan	Ishinotsubo, Nirasaki, Yamanashi	43	4030	Impression	1	7.00		Nakayama and Uruma (2014a)
Japan	Nozoe, Miyakonojo, Miyazaki	74	3830	Carbonized seed	83	4.90		Obata (2011)
Japan	Kaminabe, Kumamoto, Kumamoto	69	3500	Impression	1	7.20		Obata *et al.* (2007)
Japan	Ishinomoto, Kumamoto, Kumamoto	68	3435	Impression	1	5.30		Obata *et al.* (2007)
Japan	Kannonji-Homma-I, Kashihara, Nara	57	3035	Carbonized seed	13	6.88	1.03	Palynosurvey (2013b)
Japan	Kamisato, Kyoto, Kyoto	56	3024	Carbonized seed	10	5.23	0.53	Nishimoto *et al.* (2012)
Japan	Mitani, Tokushima, Tokushima	59	2604	Carbonized seed	22	5.99	0.62	Nasu *et al.* (2018)
Japan	Higashi-daimonsaki-II, Saku, Nagano	28	2525	Carbonized seed	113	6.11	0.68	Palynosurvey (2010)
Japan	Nagayatsu, Annaka, Gunma	14	2400	Carbonized seed	100	6.05	2.05	Sasaki and Bhandari (2012)
Japan	Saterahara, Hita, Oita	64	2400	Carbonized seed		4.10	1.27	Sasaki and Bhandari (2011)
Japan	Gangetsu, Kurihara, Miyagi	9	2300	Carbonized seed	6	5.51	0.44	Kokawa (1980)
Japan	Asada-fumbo-gun, Yamaguchi, Yamaguchi	63	2300	Carbonized seed	27	5.43	0.57	Utsunomiya (1983)
Japan	Karakami, Iki, Nagasaki	66	2275	Carbonized seed	10	4.45	0.48	Takamiya (2008)
Japan	Aoya-kamijichi, Tottori, Tottori	60	1725	Carbonized seed		5.40		Tsuji *et al.* (2011)
Japan	Nakadakase-kannonyama, Tomioka, Gunma	15	1575	Carbonized seed		5.55	0.12	Yoshikawa (1995)
Japan	Nishihiru, Matsuzaka, Mie	53	1062	Carbonized seed	23	4.95	0.55	Sasaki and Yoneda (2008)
Japan	Shimura-shiroyama, Itabashi, Tokyo	19	999	Carbonized seed	82	4.50	1.56	Palynosurvey (2012)
Japan	Nakatai, Aomori, Aomori	2	957	Carbonized seed	13	4.65	2.33	Sasaki and Bhandari (2009a)
Japan	Ueno, Aomori, Aomori	3	957	Carbonized seed	13	6.85	1.20	Sasaki and Bhandari (2010a)
Japan	Funato, Daisen, Akita	8	567	Carbonized seed	26	5.38	0.59	Palynosurvey (2014)
Japan	Maekawa, Inakadate, Aomori	5	558	Carbonized seed	15	6.10		Tsubakizaka (2009)
Japan	Kusado-sengencho, Fukuyama, Hiroshima	62	495	Carbonized seed	72	7.00		Palynosurvey (1995)
Japan	San-eicho, Shinjyuku, Tokyo	21	215	Carbonized seed		6.00		Palynosurvey (2013a)
Japan	Shibasakicho-nichome, Taito, Tokyo	20	176	Carbonized seed	58	5.40	1.13	Bhandari and Sasaki (2009)

*^a^* Site No.: Site No. appeared in the maps of Figs. 5 and 7.

**Table 2.** Soybean seeds (carbonized or impression) found in archaeological sites of China, Korea, and Japan

Country	Locality of archaeological site	Site No.*^a^*	Age Median (calBP)	Type of archaeobotanical finds	No. of samples	Mean seed length (mm)	SD of mean seed length	References
China	Jiahu, Henan	75	8800	Carbonized seed	248	3.14	0.57	Lee *et al.* (2011)
China	Dahecun, Henan	83	5400	Carbonized seed	123	3.00	0.34	Lee *et al.* (2011)
China	Xipo, Henan	77	5350	Carbonized seed	5	2.28	0.69	Lee *et al.* (2011)
China	Huizui, Henan	78	5100	Carbonized seed	10	2.12	1.02	Lee *et al.* (2011)
China	Zhaocheng, Henan	79	5100	Carbonized seed	4	1.65	0.07	Lee *et al.* (2011)
China	Zhouyuan, Shaanxi	76	4500	Carbonized seed	20	4.77	0.64	Lee *et al.* (2011)
China	Huizui, Henan	78	4500	Carbonized seed	16	3.28	1.20	Lee *et al.* (2011)
China	Wangchenggang, Henan	81	4500	Carbonized seed	20	4.65	0.31	Lee *et al.* (2011)
China	Xijingcheng, Henan	82	4500	Carbonized seed	2	4.50	0.28	Lee *et al.* (2011)
China	Shantaisi, Henan	84	4500	Carbonized seed	34	2.63	0.45	Lee *et al.* (2011)
China	Liangchengzhen, Shandong	86	4250	Carbonized seed	11	3.57	1.18	Lee *et al.* (2011)
China	Huizui, Henan	78	3800	Carbonized seed	14	4.19	0.84	Lee *et al.* (2011)
China	Yiluo valley, Henan	80	3800	Carbonized seed	4	4.40	0.70	Lee *et al.* (2011)
China	Wangchenggang, Henan	81	3300	Carbonized seed	6	5.17	0.94	Lee *et al.* (2011)
China	Daxingzhuan, Shandong	85	3300	Carbonized seed	28	2.79	1.08	Lee *et al.* (2011)
China	Zhouyuan, Shaanxi	76	3100	Carbonized seed	9	4.66	0.40	Lee *et al.* (2011)
China	Xijingcheng, Henan	82	2000	Carbonized seed	20	5.34	0.52	Lee *et al.* (2011)
Korea	Pyeonggeodong, South Gyeongsang	89	4745	Carbonized seed	19	3.22	0.36	Lee *et al.* (2011)
Korea	Nam River valley, South Gyeongsang	90	3425	Carbonized seed	9	5.97	1.27	Lee *et al.* (2011)
Korea	Daundong, Ulsan	88	2605	Carbonized seed	278	7.43	0.91	Lee *et al.* (2011)
Korea	Nam River valley, South Gyeongsang	90	2125	Carbonized seed	54	6.52	0.70	Lee *et al.* (2011)
Japan	Oujiyama, Miyakonojo, Miyazaki	73	13325	Impression	1	3.80		Obata and Manabe (2012)
Japan	Kamikurechi, Fuji-Yoshida, Yamanashi	35	9475	Impression	1	6.80		Nakayama and Shinohara (2013)
Japan	Yamanokami, Omachi, Nagano	27	9250	Impression	1	7.00		Nakazawa (2009)
Japan	Misaka-nakamaru, Fuefuki, Yamanashi	36	8400	Impression	1	7.60		Nakayama (2014a)
Japan	Tenjin, Hokuto, Yamanashi	46	5850	Impression	2	6.80	0.00	Nakayama *et al.* (2009)
Japan	Ishinotsubo, Nirasaki, Yamanashi	43	5535	Impression	1	4.30		Nakayama and Uruma (2014a)
Japan	Uenodaira, Hokuto, Yamanashi	39	5425	Impression	1	9.50		Nakayama (2014b)
Japan	Imojiya, Minami-Alps, Yamanashi	40	5180	Impression	1	9.80		Nakayama and Hosaka (2014)
Japan	Ishinotsubo, Nirasaki, Yamanashi	43	5180	Impression	1	8.50		Nakayama and Uruma (2014a)
Japan	Meotoishi, Nirasaki, Yamanashi	44	5180	Impression	1	9.00		Nakayama (2009)
Japan	Sakenomiba, Hokuto, Yamanashi	45	5180	Impression	1	6.10		Nakayama (2010)
Japan	Sakenomiba, Hokuto, Yamanashi	45	5040	Impression	1	11.80		Nakayama *et al.* (2008)
Japan	Suwahara, Hokuto, Yamanashi	47	4990	Impression	1	10.20		Nakayama and Sano (2014a)
Japan	Chikuu-I, Hokuto, Yamanashi	48	4990	Impression	1	9.80		Nakayama and Sano (2014b)
Japan	Mekiri, Okaya, Nagano	29	4955	Impression	10	9.76	1.28	Aida *et al.* (2012)
Japan	Mekiri, Okaya, Nagano	29	4955	Carbonized seed	2	2.65	0.21	Aida *et al.* (2012) Nasu *et al.* (2015a)
Japan	Shimoyakebe, Higashimurayama, Tokyo	18	4933	Carbonized seed	13	6.95	0.88	Lee *et al.* (2011) Kudo and Sasaki (2010)
Japan	Katsusaka, Sagamihara, Kanagawa	22	4855	Impression	23	4.37	0.55	Nakayama and Sano (2015)
Japan	Ishinotsubo, Nirasaki, Yamanashi	43	4855	Impression	1	9.70		Nakayama and Uruma (2014a)
Japan	Yamazaki-IV, Hokuto, Yamanashi	49	4855	Impression	2	8.90	0.28	Nakayama and Sano (2014c)
Japan	Nishikawa, Hokuto, Yamanashi	50	4855	Impression	1	5.50		Nakayama and Sano (2014d)
Japan	Miyaone, Hokuto, Yamanashi	51	4855	Impression	1	6.20		Nakayama and Sano (2014b)
Japan	Okidono, Nirasaki, Yamanashi	42	4730	Impression	1	9.00		Nakayama (2014c)
Japan	Ooyocomichiue, Hara, Nagano	31	4671	Carbonized seed	13	5.76	1.61	Nasu *et al.* (2015a)
Japan	Sakuramachi, Koyabe, Toyama	26	4643	Carbonized seed	1	8.25		Yamada and Tsubakizaka (2009)
Japan	Okidono, Nirasaki, Yamanashi	42	4615	Impression	1	9.00		Nakayama (2014c)
Japan	Meotoishi, Nirasaki, Yamanashi	44	4615	Impression	1	10.70		Nakayama (2009)
Japan	Okidono, Nirasaki, Yamanashi	42	4585	Impression	1	5.40		Nakayama (2014c)
Japan	Katsusaka, Sagamihara, Kanagawa	22	4565	Impression	1	9.50		Nakayama and Sano (2015)
Japan	Onohara, Shimabara, Nagasaki	70	3850	Impression	2	11.40	3.25	Obata (2011)
Japan	Mimanda, Kikuchi, Kumamoto	67	3500	Impression	1	14.30		Obata (2011)
Japan	Mimanda, Kikuchi, Kumamoto	67	3035	Impression	1	11.30		Obata (2011)
Japan	Kureishibaru, Shimabara, Nagasaki	71	3035	Impression	1	14.80		Obata (2011)
Japan	Sasai, Fukuoka, Fukuoka	65	2615	Carbonized seed	100	8.19		Obata (2011)
Japan	Isshiki-aokai, Inazawa, Aichi	52	2000	Carbonized seed	3	7.77	4.50	Niiyama (2008)
Japan	Toigahana, Kuze, Okayama	61	1825	Carbonized seed	2	7.50	0.57	Konishi *et al.* (2004)
Japan	Kamimiwa-kogiribata, Nobeoka, Miyazaki	72	1752	Carbonized seed	2	7.35	0.92	Palynosurvey (2013c)
Japan	Kyuhoji, Yao, Osaka	58	1625	Carbonized seed	4	8.33	3.77	Palynosurvey (2013d)
Japan	Ogura-haranishi, Sagamihara, Kanagawa	24	1225	Carbonized seed	1	7.40		Sasaki and Bhandari (2014)
Japan	Rokutanda, Sendai, Miyagi	10	1198	Carbonized seed	1	11.00		Hoshikawa (1984)
Japan	Nakamizo, Tsuru, Yamanashi	34	1198	Carbonized seed	1	5.00		Matsutani (1996)
Japan	Ueno, Aomori, Aomori	3	1025	Carbonized seed	1	8.10		Sasaki and Bhandari (2010a)
Japan	Ougita, Hirosaki, Aomori	6	1000	Carbonized seed	2	5.20	3.11	Sasaki and Bhandari (2010b)
Japan	Ju-sammori, Goshogawara, Aomori	4	925	Carbonized seed	2	8.46	0.36	Paleolabo (2013)
Japan	Jingaminejo-ato, Aizubange, Fukushima	13	800	Carbonized seed	100	9.94	1.23	Paleoenvironment-Research-Institute (2005)
Japan	Kagiage-takaoka-minami, Saitama, Saitama	16	553	Carbonized seed	2	9.65	0.07	Sasaki and Bhandari (2009b)
Japan	Hakodate-ato, Shibata, Niigata	12	450	Carbonized seed	2	6.50	0.71	Niiyama (2006)
Japan	Iwatsukijo-ato, Saitama, Saitama	17	314	Carbonized seed	1	5.50		Sasaki and Bhandari (2013)

*^a^* Site No.: Site No. appeared in the maps of Figs. 5 and 7.
